# Between life and death: strategies to reduce phototoxicity in super-resolution microscopy

**DOI:** 10.1088/1361-6463/ab6b95

**Published:** 2020-02-14

**Authors:** Kalina L Tosheva, Yue Yuan, Pedro Matos Pereira, Siân Culley, Ricardo Henriques

**Affiliations:** 1 MRC Laboratory for Molecular Cell Biology, University College London, London, United Kingdom; 2 ITQB-NOVA, Oeiras, Portugal; 3 The Francis Crick Institute, London, United Kingdom; s.culley@ucl.ac.uk; r.henriques@ucl.ac.uk

**Keywords:** phototoxicity, photodamage, super-resolution microscopy, fluorescence

## Abstract

Super-resolution microscopy (SRM) enables non-invasive, molecule-specific imaging of the internal structure and dynamics of cells with sub-diffraction limit spatial resolution. One of its major limitations is the requirement for high-intensity illumination, generating considerable cellular phototoxicity. This factor considerably limits the capacity for live-cell observations, particularly for extended periods of time. Here, we give an overview of new developments in hardware, software and probe chemistry aiming to reduce phototoxicity. Additionally, we discuss how the choice of biological model and sample environment impacts the capacity for live-cell observations.

## Introduction

The spatial resolution of an imaging system is defined as the capacity to distinguish closely separated features; in light microscopy, this is limited by diffraction to ~200–300 nm. Consequently, microscopy approaches developed to achieve resolutions beyond this limit are termed ‘super-resolution microscopy’ (SRM) [1]. SRM techniques that have recently gained popularity, such as photoactivated localisation microscopy (PALM) [2], stochastic optical reconstruction microscopy (STORM) [3], structured illumination microscopy (SIM) [4] and stimulated emission depletion (STED) microscopy [5], have enabled biological discoveries inaccessible to conventional microscopy [6–9]. Alongside increased spatial resolution, SRM retains many desirable features of light microscopy techniques, including molecule-specific labelling and the potential for live-cell imaging, unavailable to other high-resolution techniques, such as electron microscopy. However, the live-cell imaging potential of SRM has remained largely untapped as the requirements of most SRM techniques pose various challenges for exploring dynamic processes under physiological conditions. In contrast, such limitations are absent when using fixed specimens.

**Figure 1.**
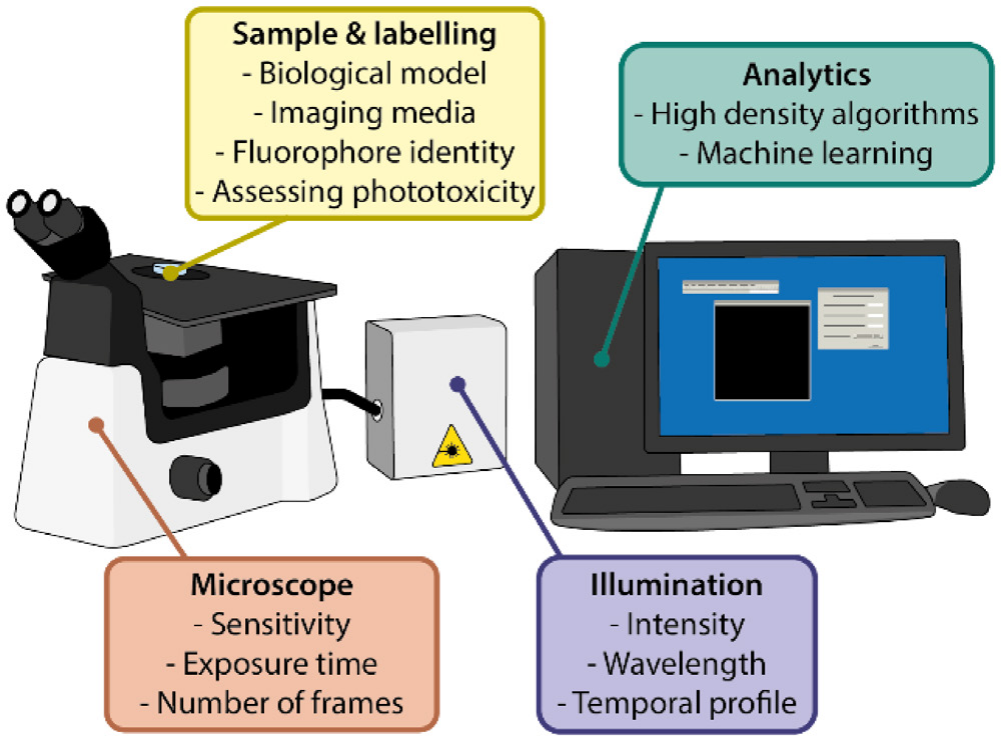
Summary of the factors that can be optimised to reduce phototoxicity in SRM.

Resolution increase in SRM is generally achieved at the cost of high-intensity illumination [10]. These requirements result in photobleaching, defined as irreversible loss of fluorescence during imaging. However, of greater importance to live-cell imaging is sample health. Thereby, the dependency of SRM on illumination intensities orders of magnitude higher than conventional microscopy (W cm^−2^–GW cm^−2^ compared to mW cm^−2^–W cm^−2^) makes phototoxicity the biggest concern when employing these techniques [10, 11]. In the context of microscopy, phototoxicity is a broad term encompassing physical and chemical reactions caused by the interaction between light and cellular components, with detrimental effects on the latter [12, 13]. Correct biological interpretations from live-cell imaging can only be achieved if the observed phenomena progress with minimal perturbation [14]. A multitude of properties of the sample and the imaging can influence phototoxicity and can thus be optimised for improving SRM for live-cell imaging (figure 1).

**Figure 2.**
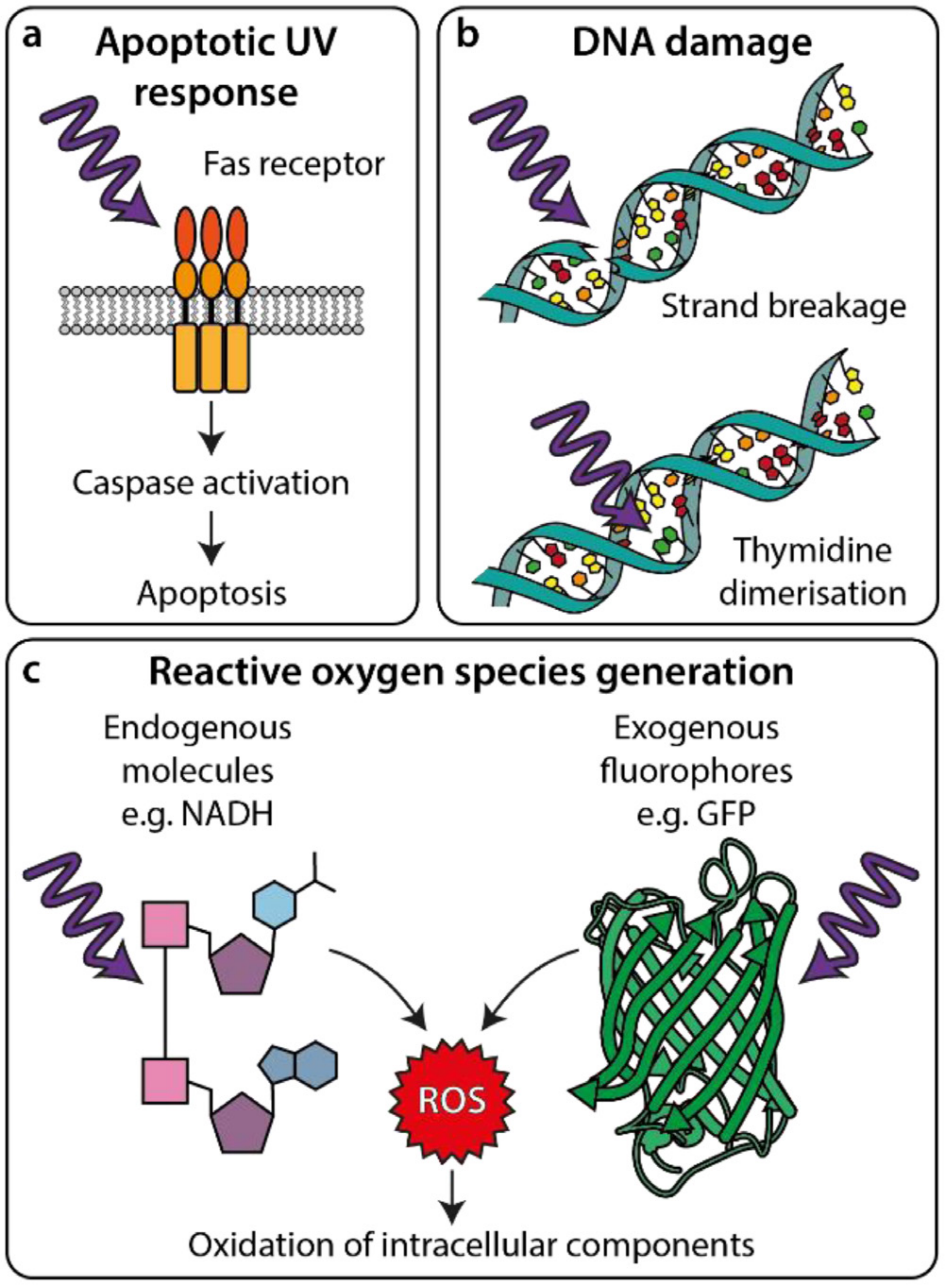
Interactions of light with cellular components leading to phototoxicity. (a) UV light can trigger apoptosis by inducing Fas receptor-mediated signalling pathways. (b) UV light can directly damage DNA by causing strand breakage (top) or thymidine dimerisation (bottom), causing mutations and inducing DNA damage responses. (c) UV and visible wavelengths can excite photoactive molecules leading to chemical generation of ROS, which can then damage cellular components.

On a molecular level, the main causes of phototoxicity are photochemical processes that directly damage intracellular components or lead to the production of toxic products within the cell or in its direct environment [15, 16]. The detrimental effects of ultraviolet (UV) light on cells is particularly well characterised; illumination with UV light can trigger the so-called ‘UV-response’ (figure 2(a)) [17, 18], DNA-strand breaks [19, 20], and thymidine dimerisations [21] (figure 2(b)), leading to mutations and downstream apoptosis [22, 23]. Additionally, both UV and visible wavelengths can excite other endogenous photoactive molecules in the cell, such as NAD(P)H [24], flavins [25, 26] and porphyrins [27, 28]. Furthermore, in fluorescence microscopy there are phototoxic effects associated with the fluorescent molecules required for labelling structures [15, 29]. Upon illumination, both endogenous and exogenous photoactive molecules can be excited to reactive states (most commonly long-lived triplet states) capable of undergoing redox reactions that lead to formation of reactive oxygen species (ROS) (figure 2(c)). ROS are considered the major contributors to phototoxicity [12, 13]. Their production can occur via direct reaction between the excited molecule and environmental molecular oxygen or via reactions with other neighbouring molecules that generate free radicals [30]. ROS have a broad range of negative effects ranging from oxidising proteins, lipids, and DNA, as well as systematic effects such as disrupting the redox homeostasis, signalling pathways and cell cycle [12, 31]. Notably, ROS production correlates with illumination intensity and photobleaching [12, 15], both of which are issues present in SRM. As a result, there is considerable interest in developing SRM technologies for improved sample health. Here, we will outline the progress in hardware, software and probe development as well as choices in biological model and sample preparation that can help improve live-cell SRM (figure 1).

## Quantifying phototoxicity in microscopy

Measuring phototoxicity in microscopy is not a trivial problem, as evidenced by the sparsity of the available literature [12, 13]. This is not entirely surprising, as phototoxicity is mediated by many factors (figure 1). These include illumination wavelength, intensity and duration of illumination, the illumination regime (e.g. LED illumination versus laser illumination, laser-scanning versus light-sheet), and the number of imaged 3D-planes [32–37]. Additionally, illumination tolerance can vary substantially between specimens (see Biological models and sample preparation section), and experimental stress can influence a specimen’s sensitivity to illumination [14]. For example, a procedure as routine as transfection or the addition of a drug has been shown to dramatically increase cellular sensitivity to light [10, 38]. Therefore, steps must be taken to reduce avoidable experimental perturbations which can influence the well-being of the sample in an illumination-independent manner, e.g. suboptimal environmental conditions (temperature, pH, etc) [39] or complex sample mounting.

How does one approach a problem as versatile as measuring phototoxicity? An intuitive and common way of assessing photodamage is by quantifying photobleaching [40–43]. However, phototoxicity and photobleaching are two separate processes; while toxic ROS are produced during photobleaching, they can also be generated independently of this process [15, 44]. Therefore, phototoxicity can commence prior to a detectable reduction in fluorescence, making photobleaching an unreliable read-out for photodamage in the context of live-cell imaging [12]. More importantly, photobleaching rates give no information on the health and viability of the specimen. Thus, a better phototoxicity measure would have a read-out related to the properties of the sample itself, rather than the properties of the fluorescence [34].

**Figure 3.**
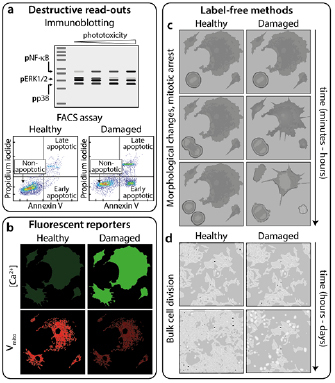
Methods for measuring phototoxicity. (a) ‘Destructive read-outs’ are techniques prohibiting further imaging of the sample. These include blotting for phosphorylated forms of proteins present in damage-activated pathways [51] and flow cytometry for determining the population of cells expressing, for example, apoptotic markers such as annexin V. (b) ‘Fluorescent reporters’ are additional indicators added to the sample during imaging whose fluorescence signal changes in response to e.g. intracellular Ca^2+^ concentration (top) or mitochondrial membrane potential (bottom). ‘Label-free methods’ of quantifying phototoxicity involve: (c) short-term observation of cell division and morphology and (d) proliferation of cells in culture following imaging. Reproduced from [51]. CC BY 4.0.

There are several *in vitro* assays for post-imaging assessment of the health and viability of a specimen that can be used to indicate whether phototoxicity occurred (figure 3(a)). These include detection of toxic ROS, fragmentation and oxidation of DNA strands, reduced metabolic activity, loss of membrane integrity and the expression of stress- and apoptosis-related proteins [45–50]. The advantages are that these assays provide an inexpensive and simple specimen viability evaluation. Thus, different illumination conditions can be tested and viability can be assessed each time. However, for such assays the measurement is limited to a single timepoint and imaging cannot be recommenced after performing the assay.

A more dynamic and practical approach entails monitoring changes in relevant biological parameters during imaging (figures 3(b) and (c)). Cellular processes which are particularly photosensitive (i.e. rapidly perturbed by light) are excellent read-outs. For example, a commonly employed method is measuring changes in cytosolic calcium concentration using calcium-sensitive fluorescent probes [50, 52–54] (figure 3(b), top). This strategy was used to evaluate live-cell STED microscopy by monitoring differences in intracellular calcium concentration between control cells and STED-imaged cells. The method showed that while there is little difference between calcium concentration in control and STED-imaged cells when using excitation and STED-lasers with wavelengths  >600 nm, responses indicative of cell damage were observed with shorter illumination wavelengths and when longer STED-laser dwell times were used [29]. Other processes exist that make suitable read-outs for phototoxicity, including changes in mitochondrial membrane potential [41, 51] (figure 3(b), bottom), reduction of chromosome movement [55] and slowing of microtubule growth [10]. It is worth highlighting that, regardless of the process chosen, care must be taken when employing fluorescent probes for visualising these read-outs [46, 56].

There are image-based phototoxicity measurements that can be performed without fluorescent labels. These often rely on identifying changes in cell morphology indicative of entry into apoptosis, such as blebbing or cell rounding [10, 14, 51, 57], for example by using transmitted light imaging (figure 3(c)). This approach was recently used to train a deep convolutional neural network, referred to as ‘DeadNet’, with the objective to automate phototoxicity detection and quantification from transmitted light images [58]. However, despite widespread use, relying on morphology as a read-out has two limitations: first, even experienced researchers can struggle to identify subtle changes in morphology, thus biasing the results (e.g. by annotating ambiguous cases incorrectly [58]; second, when changes become obvious, they usually represent an extreme phenotype indicative of irreversible damage. Thus, they cannot account for early damage that may arise even as cells display a healthy morphology [13, 39].

In this context, a read-out that deserves special mention is cell division (figures 3(c) and (d)): a well-characterised biological process with easily identifiable phases. It is highly regulated and sensitive to various perturbations, including illumination and changes in ROS concentrations [15, 31]. This makes cell cycle an excellent read-out for detection and quantification of phototoxicity [39], with both continuous (figure 3(c)) and endpoint (figure 3(d)) measurements possible. Delay in mitotic progression has been used successfully to detect perturbations in the health of both cultured cells and developing embryos [32–35]. Additionally, evaluating colony formation or number of cell divisions after illumination (typically assessed after a period of one or more cell cycles) can be indicative of long-lasting damage [12, 29] (figure 3(d)). This approach was used to perform extensive characterisation of photodamage under illumination conditions commonly used in single-molecule localisation microscopy (SMLM) [10]. The viability of several different cell lines was determined 20–24 h post illumination, a strong correlation between shorter illumination wavelengths and increased cell death was shown, particularly at high intensities. However, results also suggested that long-term cell viability is possible even with illumination wavelengths as short as 405 nm, provided the integrated light dose is small, preferably with continuous rather than pulsed illumination. Naturally, a limitation exists in employing these methods to assess phototoxicity in post-mitotic systems, e.g. primary neuron cultures. However, for relevant models, choosing mitosis as a read-out has the significant advantage of allowing phototoxicity assessment based on label-free transmitted light images [10, 29, 33], minimising the introducing additional damage during evaluation.

From reports of phototoxicity in literature, several conclusions can be drawn to guide live-cell friendly SRM. Firstly, red-shifted wavelengths are preferable to shorter wavelengths. In particular, UV wavelengths should be avoided wherever possible [10, 29, 33]. Furthermore, several studies demonstrate that lower intensity illumination with longer exposure is less damaging than short intense bursts or pulses of illumination [10, 34, 40]. Most importantly, a recurrent message throughout the literature is that higher illumination intensities are more damaging than corresponding imaging conditions with lower illumination intensities. We anticipate that real-time phototoxicity measurements will become commonplace in both diffraction-limited microscopy and SRM, and that future SRM techniques will be accompanied by a thorough description of how they impact living samples. Concomitantly, for SRM users, awareness of strategies for minimising phototoxicity is crucial.

## Fluorescent probe development for live-cell SRM

SRM techniques have distinct requirements for fluorescent probes. SIM quality relies on collecting images of high signal-to-noise ratio (SNR), generally achieved by labelling with fluorophores of high brightness and resistance to photobleaching. In STED, fluorophores must not only be bright but also possess a large Stokes-shift and stimulated emission cross-section at the STED wavelength [59]. SMLM techniques have the most demanding labelling requirements—fluorophores must be capable of cycling between ‘on’ and ‘off’ states with appropriate kinetics, a high quantum yield in the on-state, and a very low quantum yield in the off-state.

Several fluorophores and probes have been developed specifically for SRM [60, 61]. However, while many specialised fluorophores exist for fixed specimens [62], there are far fewer options available for live-cell imaging. An inappropriate choice of fluorophore for live-cell SRM will not only lead to low quality images downstream [63], but also inevitably impact acquisition settings and hence phototoxicity [10, 64].

**Figure 4.**
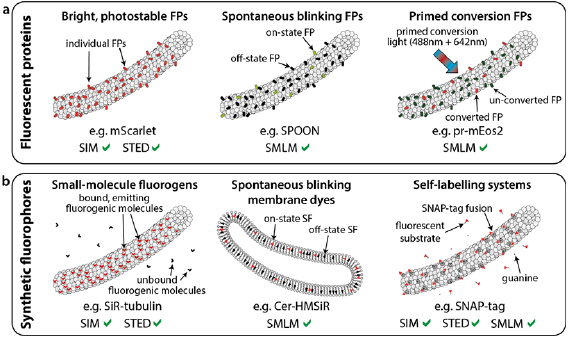
Low phototoxicity fluorescent probes and labelling for live-cell SRM. Various recently-developed fluorescent protein (a) and synthetic fluorophore (b) based methods for labelling in live-cell super-resolution. All labels are shown attached to a microtubule as an example of an intracellular structure, with the exception of the Cer-HMSiR membrane dye in (b).

As for most fluorescence microscopy techniques, the two classes of fluorophores used in SRM are fluorescent proteins (FPs) (figure 4(a)) and synthetic fluorophores (SFs) (figure 4(b)). FPs are the usual choice for live-cell imaging as they can be fused to a target of interest via genetic encoding, but at the cost of reduced brightness compared to SFs. The recent development of bright and photobleaching-resistant FPs has expanded the options for SIM and STED (figure 4(a), left). Examples of these new FPs are mNeonGreen (*λ*_ex_  =  506 nm) [65], mScarlet (*λ*_ex_  =  569 nm) [66] and mGarnet (*λ*_ex_  =  598 nm) [67]. SMLM techniques generally require photoswitchable fluorophores (e.g. mEos3.2, rsKame) [68, 69]. Despite the availability of several photoswitchable FPs, their use in live-cell imaging remains challenging [10, 64]. The chief reason is that transitions between off- and on-states are typically modulated by UV illumination. The combination of this with high intensity excitation for detection of molecular positions results in a short window for live-cell SMLM studies. To reduce phototoxicity in SMLM, FPs that do not require UV pumping for photoswitching are being developed (figure 4(a), centre), with one such example being SPOON [70]. Primed conversion is another promising UV-independent approach to induce photoswitching (figure 4(a), right) [71]. Thereby a combination of blue and near-infrared illumination induces photoconversion in Dendra2 and the newly developed primed-conversion protein pr-mEos2 [71, 72]. Recently, a general mechanism for primed conversion was described, which is anticipated to accelerate the development of more FPs that can be photoconverted with this live-cell friendly approach [73]. FPs for other specific SRM techniques have also been developed (e.g. Skylan-NS for non-linear SIM or GMars for REversible Saturable/switchable OpticaL Fluorescence Transitions, RESOLFT) [74, 75].

The second alternative, SFs (figure 4(b)), are small chemically synthesised probes. These have higher quantum yields and are more robust against photobleaching than FPs [76–79]. While there are some cell-permeable SFs that can be used to label specific proteins (e.g. fluorogens such as SiR-tubulin and SiR-actin) (figure 4(b), left) [80, 81] or cell compartments directly (e.g. Membright, ER-Tracker or MitoTracker) (figure 4(b), centre) [77, 82–84], additional `linker' molecules are normally required to associate SFs with the structure of interest. These linkers must bind the target structure with high affinity and specificity (e.g. antibodies and DNA/RNA scaffolds, usually using amine- or thiol-reactive derivatives of the SF) [85]. However, many of these high-affinity linkers and SFs are not cell-permeable, which limits their use in live-cell SRM to labelling of cell-surface molecules. If genetic encoding is possible and preferable, cell-permeable SFs can be combined with flexible self-labelling systems, such as SNAP-tag, Halo-tag or FlAsH (figure 4(b), right) [86–89]. An elegant example of such an approach is the use of Cox8A-SNAP fusion labelled with SNAP-Cell SiR for STED. This has enabled the visualisation of the dynamics of mitochondrial cristae with ~70 nm resolution [90].

SFs have also been engineered for live-cell SRM. Spontaneously blinking synthetic fluorophores (e.g. HMSiR) have been recently developed (figure 4(b), center). They do not require UV irradiation or cytotoxic additives (such as thiol) to induce photoswitching [91, 92]. High photostability SFs have also been developed, enabling live-cell STED [79, 93–95].

A final regime for live-cell SRM-compatible labelling is based on site-specific conjugation of fluorophores to a target of interest, through genetic code modifications and click chemistry (figure 4(b), right) [96–98]. These approaches combine the benefits of site-specific labelling (as is the case for FPs) with no requirement for protein expression and bright labels (as is the case for SFs).

## Biological models and sample preparation

Care should be taken when selecting a biological model for SRM. Cellular sensitivity to light exposure can vary based on cell type and species [10, 14, 45], and in the case of whole organisms, developmental stage [13, 34]. Phototoxicity has been documented for different cell types, ranging from primary cells [13, 45] to various immortalised cell lines [10, 26, 38, 99]. One such study focuses on immortalised cell lines, where it shows that COS-7 and U2OS cells exhibit similar photosensitivity, whereas HeLa cells are substantially more robust, potentially making the latter a more suitable system for live-cell SRM studies [10]. Another study illustrated the effect of photodamage on primary cells from rat central nervous system [45]. Here, illumination with blue light could induce morphological changes, differentiation or cell death depending on the cell type.

When imaging whole organisms, earlier developmental stages from the same species tend to be more photosensitive than later [12]. Furthermore, different model organisms display variable photosensitivity. For example, fruit fly embryos and nematode worms have higher illumination tolerances than zebrafish embryos, corals or cultured cells [13, 14]. Even within the same cell, different intracellular structures exhibit different responses to illumination [29, 100].

Photodamage can be mitigated through additional sample preparation steps. Established strategies centre on preventing photobleaching by modifying the sample environment. As photobleaching can contribute to phototoxicity via ROS production [44], strategies to reduce photobleaching could also help ameliorate phototoxicity [15, 29, 101]. One strategy is to modify the environmental conditions prior to or during imaging. A prime example is removal of oxygen, the main effector of photobleaching [102], from the culture medium. This can be achieved by bubbling nitrogen through the medium during imaging. This yields an increased photostability [103, 104] and, since oxygen is directly involved in the production of ROS, also reduces light-dependent oxidative stress on the sample. It has also been shown that growing cells in a hypoxic environment (3% oxygen) yielded a 25% increase in mitosis entry after blue light irradiation [33]. Other approaches to reduce oxygen in the medium involve the addition of commercially available oxygen-scavengers such as the Oxyrase^®^ enzyme complex (developed by Oxyrase, Inc., Mansfield, Ohio). In combination with suitable substrates, such as D/L-lactate or D/L-succinate, these enzymes catalytically reduce the concentration of oxygen and free radicals present in the medium, thus minimising photobleaching and phototoxicity [105, 106]. While these approaches could improve live-cell SRM, it should be noted that they are only suitable for specimens which can tolerate hypoxia or anoxia. Notably, some fluorophores used in SRM require oxygen scavenger systems to photoswitch, however, these buffers typically use cytotoxic compounds such as thiols, making them unsuitable for live-cell imaging.

A different strategy for reduction of ROS during imaging involves supplementing the media with antioxidants. Antioxidants are molecules that prevent oxidation in a biological context [107]. Among antioxidants, Trolox, the soluble form of vitamin E, has been shown to have a protective effect for a number of cell lines due to its ROS-neutralising properties [108]. The presence of the antioxidant in the sample medium has been shown to increase the number of post-illumination mitotic cells by up to 38% compared to cells illuminated without Trolox [33]. However, this molecule is not suitable for SMLM, as it has been shown to inhibit fluorophore blinking [109]. Another antioxidant used in microscopy is rutin, a plant flavonoid shown to reduce EGFP reddening [110, 111], although no direct reduction of phototoxicity was demonstrated. A notable example of a medium additive for live-cell imaging is the vitamin- and antioxidant-rich ‘supplements for optogenetic survival’ (SOS). SOS has been shown to increase viability and reduce photodamage in several cell types of the rat central nervous system [45].

There are chemicals used in mounting media, such as various antioxidants, triplet-state quenchers and radical scavengers, that can be used for photobleaching reduction and ROS neutralisation. These include ascorbic acid [112], n-propyl gallate [112–114], p-phenylenediamine [114–116], 1,4-diazobicyclo(2,2,2)-octane (DABCO) [114, 117], mercaptoethylamine (MEA) and cyclooctatetraene (COT) [112]. Their presence in mounting media for reduction of photobleaching is well characterised [112, 115, 118], however there is no comprehensive study on the use of these chemicals in live-cell imaging. As a result, there is little information regarding biocompatible working concentrations or biological side effects. Therefore, while potentially useful, they require further exploration prior to use in live-cell SRM.

Some substances commonly used as supplements are known also to cause phototoxicity, such as molecules with benzene rings which are intrinsically fluorescent [111]. For example, common cell media components, such as riboflavin and pyridoxal, can enhance oxidative reddening of GFPs; this effect accounts for a considerable part of GFP photobleaching [119]. Depleting these substances increases GFP photostability, indirectly reducing photodamage [110]. Additionally, the combination of riboflavin and tryptophan in media generates ROS and induces cytotoxicity upon illumination, whereas their removal alleviates this effect [120, 121]. Finally, the study that established the SOS supplement [45] used it in combination with the photoinert media NEUMO and MEMO, which also lack riboflavin. These media were specifically developed to prevent phototoxicity of nervous system cells. A confounding example is 4-(2-hydroxyethyl)-1-piperazineethanesulfonic acid (HEPES), commonly used as a replacement for carbon dioxide buffering during imaging [39]. However, early reports demonstrated that HEPES-buffered media exposed to low-intensity white light can generate toxic hydrogen peroxide with detrimental effects on thymocyte or T-cell culture [122, 123].

There is still a lack of information on SRM sample preparation reducing phototoxicity. Many principles can be transferred from conventional fluorescence imaging. These include assessing photosensitivity of the biological model, environmental conditions, and attention to media composition.

## Hardware developments for improved live-cell imaging

**Figure 5.**
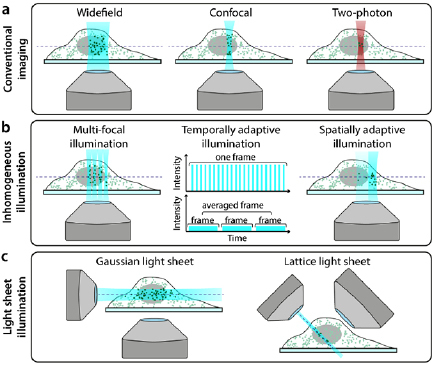
Hardware modalities for conventional and low-phototoxicity SRM. (a) Microscopy illumination regimes for conventional fluorescence imaging. (b) Examples of regimes that reduce light dose to the sample by inhomogeneous illumination. (c) Examples of light-sheet microscopy geometries.

The microscope configuration has a substantial impact on the amount of photodamage experienced by a specimen. Figure 5(a) shows the common illumination regimes for conventional microscopy and SRM (widefield for SIM and SMLM, confocal for STED). Basic optimisations of the microscope body, for example minimising photon loss in the detection path by using high-quality filters and sensitive detectors, will reduce the illumination burden to achieve suitable SNR [101]. In SRM approaches, microscopes are built with high-quality components, often having bespoke solutions to maximize signal detection [124, 125]. In addition, the ever-present phototoxic high-intensity illumination requirements of most SRM techniques can be further ameliorated using dedicated hardware designs. Interestingly, a recent study shows low-illumination live-cell SRM immediately followed by *in situ* fixation of the sample and high-illumination SRM [126]. This approach combines the collection of temporal information in living-cells with a mild resolution increase, then capture of higher resolution for a specific timepoint upon fixation.

In the case of STED, the presence of a second high-intensity laser beam (depletion laser) in addition to a confocal excitation beam confers the high phototoxicity of this method. However, the properties of both beams can have a substantial impact on sample photodamage. It has been shown in confocal microscopy that nanosecond pulsed, rather than continuous, excitation can reduce photobleaching, and that averaging multiple fast scans is less phototoxic than acquiring a single slow scan (figure 5(b), ‘temporally adaptive illumination’) [41]. The properties of the excitation beam have also been explored specifically in STED. For example, reducing the pulsing rate of the excitation laser allows time for long-lived triplet states to relax which leads to decreased photobleaching [127]. Similarly to confocal microscopy, scanning at a higher rate in STED has been shown to reduce photobleaching [42]; this is enabled by using fast resonant scanning mirrors rather than slower galvanometer scanning mirrors to scan the beam pair through the sample. Another method described reducing phototoxicity in STED is by using two-photon excitation (figure 5(a) ‘Two-photon’). As two-photon excitation only excites fluorophores within the focal volume of the beam (rather than along the entire beam path, as is the case in single-photon excitation), it is often considered a more live-cell friendly imaging regime [54, 128]. Indeed, live-cell STED has been successfully demonstrated with two-photon excitation [129, 130] although while the former paper claims that there is no photodamage to the sample, this is not quantified. It should be noted that two-photon excitation does however increase local heating, which can damage the sample in a non-fluorophore mediated manner [131].

In STED microscopy with pulsed depletion lasers, resolution scales non-linearly with beam intensity. Thus, in order to obtain high resolution images, very high (and phototoxic) depletion beam intensities are required. A different approach to obtaining high resolution STED images without this power dependence is gSTED (gated-STED) [132]. gSTED uses a continuous wave (CW) laser for the depletion beam rather than a pulsed laser. When a CW depletion beam is combined with a pulsed excitation beam, spatial information about the underlying fluorophore distribution becomes encoded in the temporal information of emission on a nanosecond timescale. By using time-gated detectors, photons detected immediately after excitation can be excluded from the final image, which improves image resolution. By tuning the size of the time-gate, gSTED can thus increase STED resolution independent of increasing light dose to the sample [133].

SIM is generally considered the least phototoxic SRM technique [134]. However, it still requires the acquisition of several frames (often  ⩾  9) at high SNR in order to generate the final reconstructed image. Several approaches have been developed to reduce the number of frames required for a SIM reconstruction, including pixel reassignment and image scanning microscopy (ISM) methods. One example is multifocal structured illumination microscopy (MSIM, [135]), which combines principles from SIM and confocal microscopy to scan an array of spots across the sample for fast live-cell imaging with resolution doubling (figure 5(b), ‘Multi-focal illumination’). Another method, rapid non-linear ISM [136], combines ISM with two-photon excitation and second-harmonic generation for low phototoxicity imaging. A wide range of such SIM-based techniques exist, and have been rigorously compared elsewhere [134, 137]. It has been demonstrated recently that using sub-millisecond pulses as excitation in SIM (when combined with novel analytics as described below) reduced photobleaching and enables long-term live-cell imaging [138].

Techniques that restrict illumination to only the focal plane of the sample are also preferable to those which illuminate along the whole beam path. One such example of this is TIRF (total internal reflection fluorescence) microscopy, where only fluorophores within a few hundred nanometers of the coverslip are illuminated. While TIRF has been combined with super-resolution modalities, such as SIM, and is effective in reducing photodamage by axially confining excitation [134], it is restrictive in that only biological structures adjacent to the cell membrane can be studied.

Light-sheet microscopy approaches similarly confine illumination to a narrow band, but their imaging geometries allow for investigation of structures throughout the whole sample and not just regions close to the coverslip. The majority of them involve illuminating the sample with a thin sheet of light and then detecting the fluorescence perpendicular to the direction of sheet propagation (figure 5(c), ‘Gaussian light sheet’) [139, 140]. This confers low phototoxicity as only the part of the sample being imaged is illuminated without the need for non-linear optical processes (which is the case in two-photon microscopy). Indeed, light-sheet microscopy was named the Nature Methods technique of the year in 2014, in part due to its low phototoxicity [141]. There are several ways in which light-sheet microscopy schemes can yield super-resolution with reduced phototoxicity. Super-resolution in live samples has been demonstrated using light-sheet microscopy by simply combining this illumination geometry with SRM techniques such as SMLM [142–144] and RESOLFT [145]. However, the employed SRM methods still require high-intensity illumination, and thus such composite techniques do not exploit the inherent low phototoxicity of light-sheet imaging. Therefore, a more elegant approach involves illuminating the sample with a light-sheet regime followed by the application of SMLM analytics designed for ultra-high-density datasets, which allows for reduction of the illumination power ([146] and *Analytics* section, see below). The more widely-explored method for combining SRM and light-sheet microscopy has been the use of novel methods for generating and shaping the light-sheet. Bessel beams have been used to generate thinner light-sheets [147], and these beams have also been extended to incorporate SIM [148]. The latter strategy has also been demonstrated on a system with two counterpropagating light-sheets formed using standard Gaussian beams [149]. The most radical and live-imaging-friendly light-sheet SRM technique developed to date is lattice light-sheet microscopy [150] (figure 5(c), ‘Lattice light sheet’). This has demonstrated 3D time-lapse super-resolution imaging in both cultured cells and intact model organisms with minimal phototoxicity.

An interesting approach to reducing the illumination dose in SRM is using spatially varying illumination depending on the structural content of the imaging region (figure 5(b), ‘Spatially adaptive illumination’). This approach was originally demonstrated for confocal imaging [48] and has since been extended to SIM [151], RESOLFT [152] and indeed light-sheet microscopy [141]. There is also a range of adaptive illumination STED techniques that have been developed [153–155], and while these predominantly focus on reducing light dose in the context of photobleaching, this will concomitantly also impact the live-cell compatibility of these techniques.

## Analytical approaches to live-cell SRM

**Figure 6.**
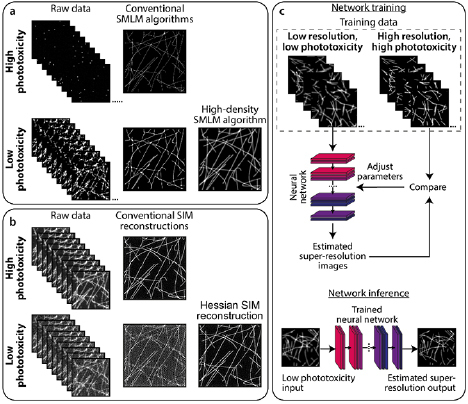
Analytics to complement low-phototoxicity imaging regimes. (a) Top: typical SMLM images are successfully reconstructed from sparse blinking raw data acquired under high phototoxic illumination. Bottom: reducing phototoxic illumination leads to more emitting fluorophores per raw data frame. When reconstructed using conventional SMLM algorithms, these produce low-quality images containing artefacts. High density SMLM algorithms can produce better quality images from such datasets. (b) Top: typical SIM imaging involves acquiring 9–25 raw images (depending on the number of grating rotations and phases) at high SNR, which can be successfully reconstructed using conventional SIM algorithms. Bottom: decreasing the illumination intensity, and thus SNR of the raw images, leads to artefacts in images reconstructed using conventional methods. The Hessian SIM deconvolution algorithm can bypass this limitation [138]. (c) Deep neural networks can be trained to infer super-resolution information from e.g. low-resolution diffraction-limited or low-quality super-resolution images. In this example, a neural network can be trained on pairs of low resolution/super-resolution images of the trained structure (‘Network training’). The trained network can then be applied to unseen low resolution images to infer the super-resolution equivalents (‘Network inference’).

Analytics can be used to extract super-resolution information from images acquired at low illumination, and thus low phototoxicity (figure 6). Such techniques are generally based on SMLM principles but improve its live-cell compatibility (figure 6(a)). In SMLM, when high intensity illumination is used, fluorophore blinking is sparse and thus the well-separated single molecules are straightforward to detect and localise with high accuracy and precision [156, 157]. However, as intensity is decreased towards a lower phototoxicity regime, blinking becomes more dense and molecules become increasingly overlapped. Such datasets require specialised algorithms to extract molecule locations. The first example of such an algorithm was super-resolution optical fluctuation imaging (SOFI), where the temporal statistics of fluorophore intensity oscillations are used to generate images with sub-diffraction resolution [158]. Indeed, SOFI has been used to image live cells [159] although only for short periods of time due to the requirement for UV illumination to induce photoswitching. Another algorithm developed for analysing datasets with dense blinking is 3B [160], where super-resolution images can be obtained from datasets imaged with a xenon arc lamp rather than lasers. However, both SOFI and 3B techniques still rely on photoswitchable fluorophores, which have drawbacks discussed above. The super-resolution radial fluctuations (SRRF) algorithm allows for the reconstruction of super-resolution images from datasets containing non-photoswitchable fluorophores such as GFP [161, 162]. SRRF has been shown to work on datasets obtained with confocal and LED-illuminated microscopes, with the latter enabling continuous live-cell imaging for  >30 min [163]. However, SRRF cannot retrieve resolutions in these regimes as high as those achievable with photoswitchable fluorophores. A promising new development for analysing high-density datasets is Haar wavelet kernel (HAWK) [164]. HAWK is a pre-processing algorithm that separates fluorophores in time; this creates an artificial lower-density dataset, which can then be analysed using any SMLM algorithm.

While most analytical developments for live-cell SRM centre on SMLM-based techniques, there are also analytics for enabling lower phototoxicity imaging in SIM and STED. Hessian-SIM is a deconvolution algorithm that can obtain high-quality SIM images from raw data acquired at low signal-to-noise ratio (figure 6(b)) [138]. This overcomes a substantial barrier in SIM, in that conventional SIM reconstruction algorithms perform poorly on low-illumination datasets, leading to artefacts within the resulting images. Approaches have also been proposed for low-power STED microscopy based on reconstructing images with knowledge of fluorescence lifetime changes induced by the STED beam [75, 165].

A rapidly evolving field in microscopy image analysis is the use of machine learning (ML)-based techniques [166, 167]. Such techniques are used for diverse applications including object segmentation, denoising, and structure prediction, and these can also be extended to SRM (figure 6(c)). One example is content aware image restoration (CARE), where a neural network is trained on high illumination intensity datasets (i.e. high phototoxicity) and used to denoise corresponding datasets acquired at much lower illumination intensities [168]. CARE was demonstrated to enhance resolution of GFP-tagged microtubules to a similar extent to SRRF analysis of the same data, but with higher quality and higher temporal resolution. There are also specialised ML algorithms for super-resolution applications. ANNA-PALM is a method that, after training a neural network with sparse SMLM data, can reconstruct super-resolution images from dense data and a correspondingly lower number of frames [169]. While not demonstrated in live-cell data, this technique could in theory alleviate phototoxicity with minimal sacrifice to spatial resolution by imaging photoswitchable FPs with lower illumination intensity. Other ML-based techniques have also allowed for prediction of enhanced resolution images from low illumination diffraction-limited images (figure 6(c)), for example: converting confocal to Airyscan-type or STED-type images [75, 170]; or widefield to SIM-type images [75].

## Discussion and outlook

High quality live-cell fluorescence microscopy involves compromising between four key properties: SNR, imaging speed, spatial resolution, and sample health [12]. We present an overview of the challenges faced on how to balance the latter two properties in live-cell SRM, highlighting potential strategies to maximise resolution while minimising phototoxicity.

As commercial super-resolution systems become commonplace in biological labs and open-source microscope hardware becomes more widespread, there is a growing desire to translate cell biology experiments from conventional diffraction-limited microscopes to higher resolution alternatives. However, the cost of this increased resolution is often the sample health. Users must be aware of what phototoxicity is, how to detect it, and methods that can be used to ameliorate it. Unfortunately, there are very few dedicated studies discussing phototoxicity specifically in SRM [10, 29].

It is clear that there are several frontiers for optimising SRM protocols for minimising phototoxicity, and a much-needed development in the field is a non-perturbing robust indicator of sample health during imaging. Caution must be taken when reporting and evaluating phototoxicity as it would also require using uniform metrics for data quality. There is already software available for assessing the quality and resolution of SRM images [171, 172]. Comparative analytics for phototoxicity would thus provide a complete numerical framework for experiment optimisation.

As super-resolution microscopes become increasingly standard equipment in biological research, users must be aware of their limitations in live-cell imaging. Many of the suggestions offered in this review for reducing phototoxicity remain under active development, and it is imperative for users to follow progress in hardware, analytics and fluorophores to ensure that they are minimising photodamage to samples.
